# Remote monitoring of amyotrophic lateral sclerosis using wearable sensors detects differences in disease progression and survival: a prospective cohort study

**DOI:** 10.1016/j.ebiom.2024.105104

**Published:** 2024-04-06

**Authors:** Jordi W.J. van Unnik, Myrte Meyjes, Mark R. Janse van Mantgem, Leonard H. van den Berg, Ruben P.A. van Eijk

**Affiliations:** a Department of Neurology, UMC Utrecht Brain Centre, University Medical Centre Utrecht, Utrecht, the Netherlands; b Biostatistics & Research Support, Julius Centre for Health Sciences and Primary Care, University Medical Centre Utrecht, Utrecht, the Netherlands

**Keywords:** Amyotrophic lateral sclerosis, Biomarkers, Clinical trials, Wearable sensors, Remote monitoring

## Abstract

**Background:**

There is an urgent need for objective and sensitive measures to quantify clinical disease progression and gauge the response to treatment in clinical trials for amyotrophic lateral sclerosis (ALS). Here, we evaluate the ability of an accelerometer-derived outcome to detect differential clinical disease progression and assess its longitudinal associations with overall survival in patients with ALS.

**Methods:**

Patients with ALS wore an accelerometer on the hip for 3–7 days, every 2–3 months during a multi-year observation period. An accelerometer-derived outcome, the Vertical Movement Index (VMI), was calculated, together with predicted disease progression rates, and jointly analysed with overall survival. The clinical utility of VMI was evaluated using comparisons to patient-reported functionality, while the impact of various monitoring schemes on empirical power was explored through simulations.

**Findings:**

In total, 97 patients (70.1% male) wore the accelerometer for 1995 days, for a total of 27,701 h. The VMI was highly discriminatory for predicted disease progression rates, revealing faster rates of decline in patients with a worse predicted prognosis compared to those with a better predicted prognosis (*p* < 0.0001). The VMI was strongly associated with the hazard for death (HR 0.20, 95% CI: 0.09–0.44, *p* < 0.0001), where a decrease of 0.19–0.41 unit was associated with reduced ambulatory status. Recommendations for future studies using accelerometery are provided.

**Interpretation:**

The results serve as motivation to incorporate accelerometer-derived outcomes in clinical trials, which is essential for further validation of these markers to meaningful endpoints.

**Funding:**

10.13039/501100014076Stichting ALS Nederland (TRICALS-Reactive-II).

**Research in context**

**Evidence before this study**

Accelerometer-derived outcomes obtained using wearable sensors could enhance the efficiency of drug development for amyotrophic lateral sclerosis (ALS), but require validation with conventional clinical outcomes to support their use in clinical trials. PubMed searches were conducted until September 2022 using the following search terms: “amyotrophic lateral sclerosis”, “ALS”, “motor neuron disease∗”, “MND”, “wearable∗”, and “acceleromet∗. While identified studies have demonstrated that outcomes derived from wearable sensors are able to describe patient-reported functional loss, little attention was given to their ability to differentiate between disease progression rates or overall survival.

**Added value of this study**

In this prospective cohort study, we determine the ability of an outcome derived from a hip-worn accelerometer to detect differential clinical disease progression and assess its longitudinal associations with overall survival in 97 patients with ALS during a multi-year observation period. We show that the outcome is highly discriminatory for predicted disease progression rates and strongly associated with overall survival. In addition, we establish that the outcome is highly related to patient-reported functional loss and ambulatory status. Finally, we identify potential clinically meaningful differences and explore the influence of different monitoring schemes on statistical power, which could provide valuable insights for the design of future studies using accelerometery.

**Implications of all the available evidence**

Utilizing digital tools like wearable sensors allows for an objective, detailed, and near-continuous collection of data in the home setting, potentially enhancing the detection of functional treatment effects. Furthermore, these technologies might ultimately offer a less burdensome, decentralised, and more patient-focused trial design. The data presented in this study serve as a motivation to incorporate accelerometer-derived outcomes in clinical trials, something which will be essential for exploring their potential as candidate surrogate markers.

## Introduction

Amyotrophic lateral sclerosis (ALS) is characterised by the progressive loss of motor neurons, resulting in significant paralysis of voluntary muscle groups, limitations in daily life and, ultimately, death within, on average, three to five years after symptom onset.^1^ Extensive clinical heterogeneity in symptom onset, progression rates and survival time is a characteristic feature of ALS and, combined with the lack of sensitive outcome measures of clinical progression, one of the main drivers of numerous futile clinical trials.^2^, ^3^, ^4^

Current outcomes for ALS clinical trials rely on subjective, mostly survey-based outcomes to assess various aspects of daily functioning and symptomatology.^5^^,^^6^ Although these outcomes have been well-adapted and successfully identified beneficial treatment effects,^7^ they have a limited ability to detect subtle changes over time.^8^ Consequently, clinical trials require large sample sizes or long follow-up durations, increasing costs and exposure to placebo, or alternatively, utilise overly restrictive eligibility criteria in an attempt to only include those patients with similar clinical progression rates. In addition, summation of the different disease domains introduces multidimensionality,^9^^,^^10^ complicating the assessment of treatment effects.^11^

Hence, the development of more sensitive and robust outcomes could enhance the efficiency of drug development. Contrarily to survey-based methods, outcomes based on digital health technology purposely assess only one disease domain (e.g., speech, motor, or respiratory function) and allow for an objective and detailed assessment, potentially improving their quantification of domain-specific disease progression.^11^, ^12^, ^13^ For the assessment of motor function, various devices have been developed,^14^ collecting data during specific exercises or obtaining data passively throughout the day. Advantages of outcomes derived from passive monitoring are that they are not bound to the patient's ability to complete a specific exercise, may have higher compliance,^15^ and may better reflect the patient's real-world functioning.^16^ As these outcomes commonly measure precise characteristics of body movements, it is not always immediately apparent what their clinical relevance is. Additional information is therefore required to facilitate meaningful interpretation and support their utility for and uptake in clinical trials.^17^^,^^18^

Previous studies have demonstrated that accelerometer-derived outcomes using passive monitoring are able to describe disease progression in patients with ALS, showing significant correlations with the ALS functional rating scale (ALSFRS-R).^19^, ^20^, ^21^, ^22^, ^23^, ^24^ Currently, however, there has been little attention to whether these outcomes accurately differentiate between rates of disease progression and fully encompass the heterogeneous nature of ALS. Moreover, although it is likely that accelerometer-derived outcomes relate to survival,^25^^,^^26^ affirming this relationship is important, as survival remains the gold standard for demonstrating therapeutic benefit^27^ and a requirement by most regulatory agencies. Additionally, while already more closely examined in other neuromuscular diseases,^28^^,^^29^ operational aspects of accelerometery, such as the required monitoring scheme, have not been fully elucidated for ALS and often remain arbitrary.^14^

Here we demonstrate that an outcome measure (Vertical Movement Index [VMI]) derived from a hip-worn accelerometer, is highly discriminatory for future disease progression and strongly associated with overall survival in patients with ALS, thereby supporting its use as a potential outcome for clinical trials. We evaluate the clinical utility of VMI through comparisons with patient-reported functional loss and ambulatory status based on the ALSFRS-R, deriving potentially meaningful differences, and explore the influence of different monitoring schemes on statistical power through simulations.

## Methods

### Participants, procedures, and devices

The data for this study originated from two prospective cohort studies performed between September 2016 and January 2023 at the University Medical Centre (UMCU), the Netherlands. Patients were included if they had a diagnosis of possible, probable (laboratory supported) or definite ALS, according to the revised El Escorial criteria,^30^ or had been diagnosed with progressive muscular atrophy (PMA) or primary lateral sclerosis (PLS), were over the age of 18 years, and were able to provide informed consent (Supplementary Table S1). Patients were recruited from the Treatment Research Initiative to Cure ALS (TRICALS) database or the biobank for motor neuron diseases of the UMCU, where patients can register to be approached for new research initiatives. The study physicians reviewed the medical records of all participating patients to confirm their diagnosis and to calculate their risk profiles according to the ENCALS survival prediction model, as described in detail elsewhere.^31^ In short, the risk profile is based on the linear predictor of the ENCALS model, which was developed for predicting individual time to death or respiratory insufficiency based on data of >11,000 patients.^32^ The resulting baseline risk profiles predict future disease progression rates and can consequently be used to quantify disease heterogeneity. Subsequently, patients were given the ActiGraph GT9X Link, which is a small, lightweight, research-grade accelerometer (cost as of 2021: $315). Patients were instructed to wear the device on the right hip in the anterior axillary line, using a belt clip, during waking hours for three to seven consecutive days, repeating the assessment every two to three months, for a maximum follow-up of eighteen to 24 months. The device was sent and returned by mail or was handed over during study visits, together with a wear time log detailing the start and stop date. In cohort two, accelerometery was part of a more extensive longitudinal study, requiring patients to visit the clinic. Follow-up visits were optional, meaning that patients were asked after each in-clinic visit whether they would like to return for another assessment. As such, patients declining further in-clinic visits were withdrawn from the study and their accelerometery assessments stopped, resulting in variable follow-up time. In addition, the ALSFRS-R was either self-administered remotely or administered by a physician during in-clinic visits. The self-administered version was validated for the Dutch population, showing excellent inter-rater reliability and acceptable inter-rater agreement between patient and clinicians.^33^ Survival status was monitored using an online nationwide population registry (data extraction in August 2023).

### Ethics

Both cohort studies were approved by the Medical Ethics Committee of the UMCU (registration numbers 16/606 and 15/656) and conducted in accordance with the granted ethical approval. All patients provided written informed consent to participate.

### Data collection and processing

During wear time, tri-axial accelerometer data were collected in free-living conditions at a sampling rate of 30 Hz. Sensor accelerations during wear periods were auto-calibrated for local gravity^34^ and compared to their expected accelerations to determine the device orientation on a day-to-day basis. Any identified discrepancies in relation to the study protocol were corrected by rotation of the axes (e.g., wearing the device upside down). Frequency filtering (a 7^th^ order IIR filter) and amplitude thresholds were used to remove or curtail accelerations probably attributable to non-human body movements.^35^^,^^36^ The raw accelerometer data were then summarised to activity counts during 10-s epochs while applying the low-frequency extension (LFE) algorithm.^36^ The LFE algorithm reduced the activity count threshold, thereby increasing the sensitivity for capturing lower intensity activities. The resulting data consisted of activity counts alongside the forward, vertical (i.e., movement against gravity), and sideway axes per 10 s. Non-wear periods were identified using the non-wear time classification algorithm reported by Choi et al.^37^ with optimised hyperparameters (periods were classified as non-wear if no activity was recorded for a minimum interval of 210 min),^38^ and these periods were subsequently removed. To obtain representative accelerometer data during a day, days with less than 8 h of total wear time were excluded from the analyses. Data of patients who participated in both cohorts were combined and treated as part of their first cohort. Survival time was defined as time from first accelerometer measurement until death or latest known follow-up. Survival time longer than 12 months since the last accelerometer measurement, or longer than 30 months since first assessment, were censored administratively.

### Vertical Movement Index

Next, the Vertical Movement Index (VMI) was derived from the processed accelerometery data. The VMI was developed to measure the patient's performance range, thereby reflecting the variability in movements induced by actions performed in an unsupervised environment. The index is based on movements against gravity by calculating the daily average variation alongside the vertical axis (standard deviation of the natural logarithmic transformation of the daily activity counts + 1). By calculating the variability in movements, VMI aims to mitigate the noise caused by between-patient differences in, for example, motivation and lifestyle. Consequently, it aims to portray the patient's ability to perform a range of actions, regardless of how often the action occurred during the day. The VMI has previously been identified as a more sensitive outcome compared to classical metrics, such as %active or Metabolic Equivalent of Task (MET), while also showing excellent correlations with the ALSFRS-R and King's staging in patients with ALS.^19^

### Sample size estimation

Our literature review found no data regarding the relationship between accelerometer-derived outcomes and overall survival in patients with ALS. However, since VMI was found to be highly correlated with the ALSFRS-R total score,^19^ sample size was calculated using the well-established relationship of ALSFRS-R with overall survival. We assumed that every point increase in ALSFRS-R total score reduces the hazard of death by 12% (HR 0.88) and that the baseline variability of the ALSFRS-R has a standard deviation of 5.^26^ In total, 30 events would provide 92% power to detect the hypothesised HR using the log-rank test with an alpha of 5%; 25 events would provide 86% power.

### Statistics

As longitudinal assessments in ALS are typically confounded by death, we analysed all data within the joint-modelling framework.^39^ In brief, the joint modelling framework consists of two parts: (1) a submodel describing the longitudinal trajectory of a clinical outcome, here the accelerometer-derived outcome VMI, and (2) a submodel that describes the event process, here time to death. By combining these models into a single framework, where the longitudinal submodel serves as a ‘covariate’ in the time-to-event submodel, one can adjust for the (informative) missing data mechanism in the longitudinal data, while investigating the predictive ability of the clinical outcome for the event. This framework was used to answer two primary questions: (1) what is the mortality-adjusted longitudinal trajectory of VMI and the ability of the baseline risk profile to predict its rate of decline, and (2) what is the relationship between VMI and the immediate rate of death (hazard).

The longitudinal submodel consisted of a linear mixed effects model, with time since first assessment in months as a fixed effect, and a random intercept and slope for time per patient as random effects. A second model was constructed by adding the baseline risk profile score and its interaction with time as fixed effects, to answer the question whether the average rate of decline in VMI over time was dependent on the baseline risk profile. For the survival submodel, the time since first assessment in months to death was modelled using a Weibull model, containing only the risk profile and longitudinal submodel as covariates. The random-effect terms followed a multivariate normal distribution. A sensitivity analysis was performed investigating four different association structures between the longitudinal submodel and the hazard (i.e., current value, current slope, current value and current slope, and history of all values until current value).^39^ Overall model fit was compared using the Akaike Information Criterion; the best fitting model (current value) was used to answer the various research questions. Similar models were used to make dynamic predictions and illustrate the ability of the current value of VMI to predict survival probabilities for individual patients, and to investigate the relationship of the ALSFRS-R total score and subdomain scores with the immediate rate of death (hazard).

Next, we evaluated the longitudinal associations between VMI and the subdomains of the ALSFRS-R. In brief, a multivariate linear mixed effect model was defined as described elsewhere.^11^ From the multivariate model, the associations between random effects were investigated using Pearson correlation analysis to understand how a change in VMI was associated with a change in each subdomain. In addition, the relationship between VMI and patient's ambulatory status based on item eight of the ALSFRS-R of that same session was assessed to determine the magnitude of VMI changes as function of symptoms reported by the patient and explore meaningful differences. Estimates of VMI per item score were modelled using linear mixed effect model with item score as fixed and random effect per patient.

Finally, to explore the influence of different monitoring schemes on statistical power, we first fitted our final model to a typical clinical trial population (i.e., patients with risk profile scores between −6.0 and −2.0).^40^ Based on the parameter estimations of this model, we then simulated clinical datasets for various monitoring schemes by varying the sampling frequency (biweekly, monthly and bimonthly) and monitoring period (1–7 days) for different study durations (6 and 12 months) and sample sizes (25 and 50) while assuming an adherence rate of 90%. In each dataset, we evaluated whether a differential progression in VMI could be detected based on the time by risk profile interaction term. We simulated each scenario 10,000 times and counted the number of simulations that resulted in a *p* value < 0.05 (empirical power). Analyses were performed using R software (version 4.3.2). Joint models were fitted using the package JM (version 1.5–2). R code for all data preprocessing is publicly available on GitHub repository (https://github.com/tricals-methodology/remote-monitoring-als).

### Role of funders

The funder of the study had no role in study design, data collection, data analyses, interpretation, or writing of the report.

## Results

### Study population and compliance

**Fig. 1:**
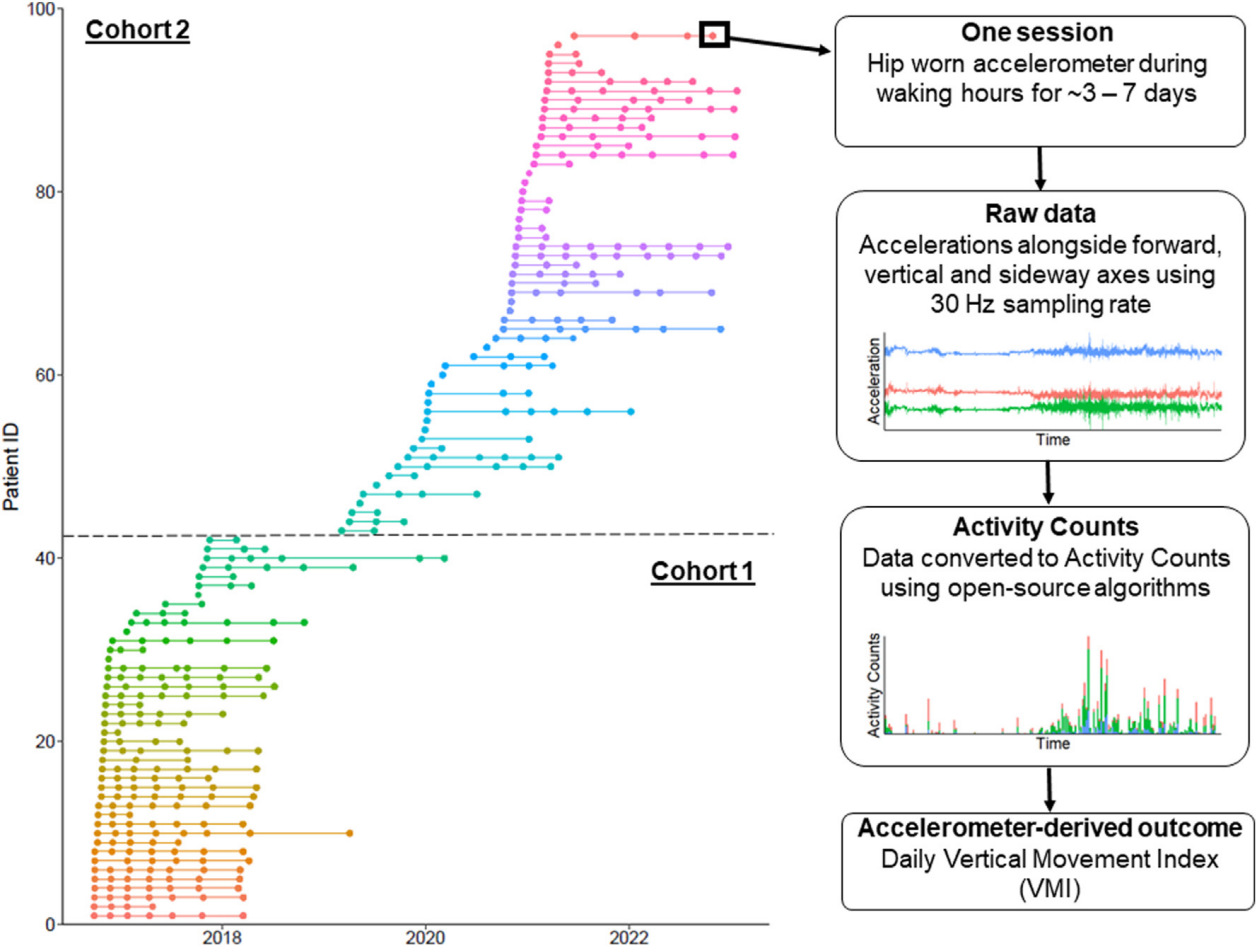
Overview of the dataset. Data collection from 2016 to 2023 along with the data extraction method and calculation of the Vertical Movement Index.

Between September 2016 and January 2023, hip-worn accelerometer data of 97 patients with ALS were collected as part of two prospective cohort studies (Fig. 1). Demographics and disease-specific characteristics are presented in Table 1. Overall, patients enrolled in the first cohort had a longer symptom duration compared to patients enrolled in the second cohort (median 24.9 vs. 18.5 months, respectively). The median follow-up for the first cohort was 17.3 [12.4] months per patient and for the second cohort, 6.9 [15.1] months per patient. The total follow-up duration encompassed 1052 patient-months with a median follow-up of 10.1 [14.6] months per patient. In total, 1969 valid wear days were available for analysis with a total monitoring period of 27,284 h and a mean daily monitoring time of 13.9 h/day. During follow-up, the mean daily monitoring time of each session decreased non-significantly from 13.6 h/day in the patient's first session to 13.4 h/day in the patient's last session (*p* = 0.92; likelihood ratio test). The wear time adherence of 91.8% was excellent (1969 ≥8-h periods of the 2145 days). A total of 32 patients died.

**Table 1:** Demographics and clinical characteristics at baseline.

	Cohort 1 (n = 42)	Cohort 2 (n = 55)	Total (n = 97)
**Demographics**			
Age (years), mean (SD)	59.9 (11.6)	61 (10.7)	60.5 (11.1)
Sex			
Male	31 (73.8)	37 (67.3)	68 (70.1)
Female	11 (26.2)	18 (32.7)	29 (29.9)
**Clinical characteristics**			
Diagnosis			
ALS	39 (92.9)	55 (100)	94 (96.9)
PMA	3 (7.1)	–	3 (3.1)
Symptom duration (months)			
Median [IQR]	24.9 [21.4]	18.5 [14.7]	22.1 [17.6]
Range	6.9–217.9	2.2–93.9	2.2–217.9
Diagnostic delay (months)			
Median [IQR]	7.5 [11.7]	11.7 [13.0]	9.6 [13.5]
Range	2.1–130.1	1.0–64.7	1.0–130.1
ΔFRS (points per months)			
Median [IQR]	0.34 [0.53]	0.37 [0.49]	0.34 [0.49]
Range	0.05–1.24	0.03–4.15	0.03–4.15
Bulbar onset	7 (16.7)	12 (21.8)	19 (19.6)
Spinal onset	34 (81.0)	43 (78.2)	77 (79.4)
Spinal arm onset	14 (41.2)	20 (46.5)	34 (44.2)
Spinal leg onset	18 (52.9)	22 (51.2)	40 (51.9)
Missing	2 (5.9)	1 (2.3)	3 (3.9)
Generalised onset	1 (2.4)	–	1 (1.0)
ALSFRS-R total score, mean (SD)	36.3 (8.1)	39.1 (5.3)	37.9 (6.8)
Bulbar subdomain	10.1 (2.7)	10.4 (1.9)	10.3 (2.3)
Fine subdomain	7.9 (3.6)	8.9 (2.9)	8.4 (3.2)
Gross subdomain	7.3 (3)	8.3 (2.7)	7.8 (2.8)
Respiratory subdomain	11.1 (1.8)	11.5 (0.9)	11.3 (1.4)
Ambulatory^a^	40 (95.2)	54 (98.2)	94 (96.9)
Only lower limb involvement^b^	–	4 (7.3)	4 (4.1)
VC %predicted (GLI-2012), mean (SD)	101.4 (16.1)	92 (16.9)	95.8 (17.1)
Missing	5 (11.9)	1 (1.8)	6 (6.2)
Riluzole use	30 (71.4)	41 (74.5)	71 (73.2)
Missing	2 (4.8)	–	2 (2.1)
Body mass index (kg/m^2^), mean (SD)	24.8 (3)	25.4 (3.3)	25.1 (3.2)
Linear predictor risk profile score, mean (SD)	−5.25 (1.79)	−5.08 (1.80)	−5.16 (1.78)

Time-related variables were summarised using median (IQR and range), mean and standard deviation (SD) for continuous variables, and frequency and percentage for categorical variables.

Abs. ALS, Amyotrophic Lateral Sclerosis; ALSFRS-R, ALS Functional Rating Scale; PMA, Progressive Muscular Atrophy; VC, Vitality Capacity; ΔFRS, 48—ALSFRS-R total score/symptom duration.

a Ambulatory defined as ALSFRS-R item 8 score of 2 or higher.

b Lower limb involvement defined as ALSFRS-R gross subdomain score of 11 or lower.

### Rate of decline and heterogeneity in disease progression between patients

**Fig. 2:**
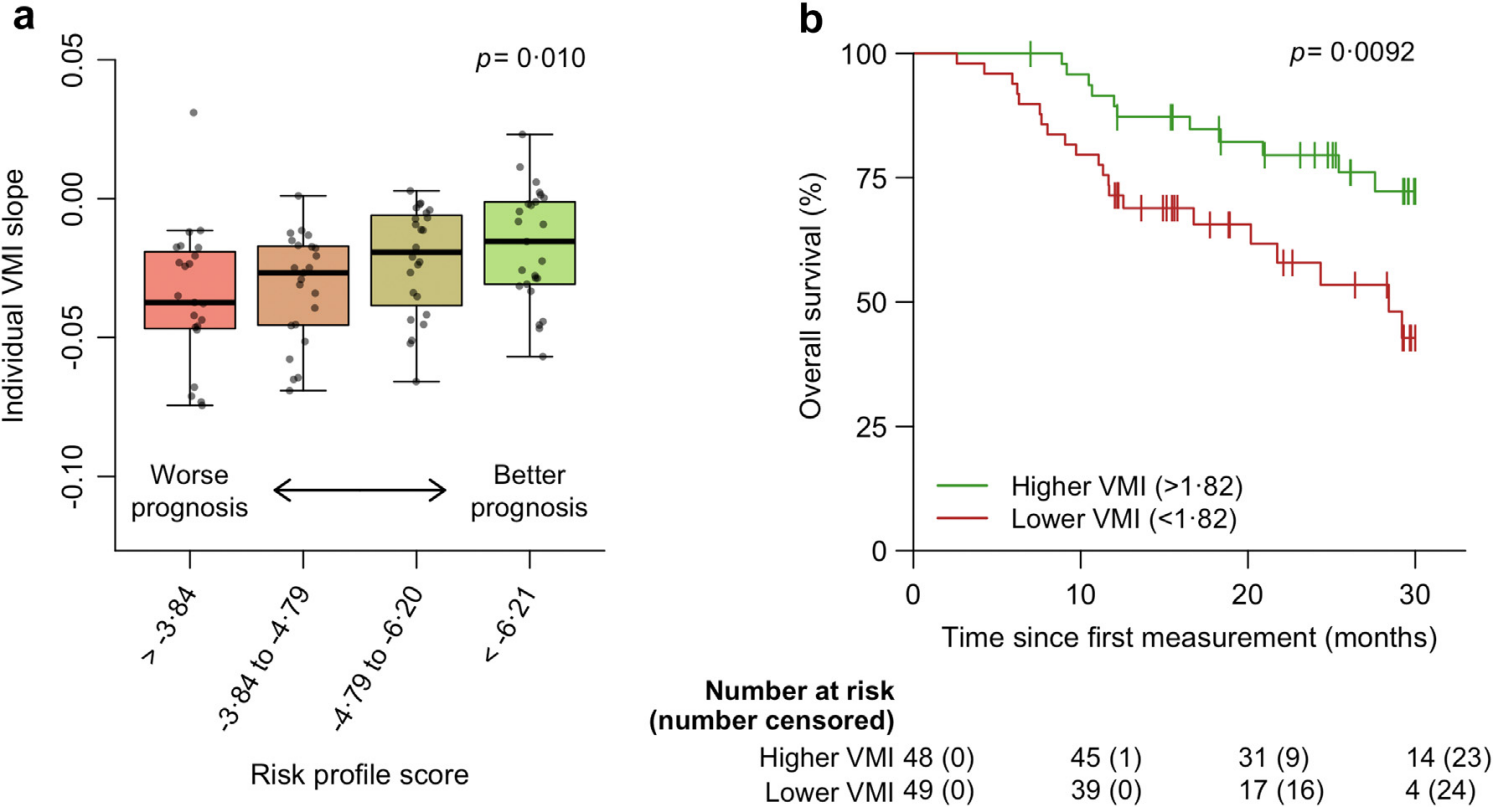
VMI heterogeneity and relationship with survival hazard. We calculated a patient-specific VMI slope from our joint model, i.e., the Best Linear Unbiased Prediction (BLUP), and correlated each BLUP to its corresponding risk profile score (predicted prognosis) to illustrate their relationship. Risk profile scores were divided into quantiles. Outliers were defined as values lower or higher than 1.5 times the interquartile range for the first quartile and third quartile, respectively. *p* value based on the one-way ANOVA (n = 95; two patients with a single wear day were excluded) (**a**). In addition, we illustrated the relationship between baseline VMI and overall survival. The VMI at baseline were divided into a “higher” and “lower” score based on their position in relation to the median. *p* value based on the log-rank test (n = 97) (**b**). Abs. VMI, Vertical Movement Index.

The VMI declined over time with an average rate of 0.028 units per month (95% CI: −0.035 to −0.020, *p* < 0.0001; Wald test). There was significant variability between patients (*p <* 0.0001; likelihood ratio test) in their rates of decline, with patient-specific slopes ranging from −0.153 to 0.024 units per months. This is of significance given the known heterogeneous nature of ALS and highlights the potential of VMI to reflect differential disease progression rates between patients. This was further explored by associating the VMI progression rate with the patient's baseline prognostic risk profile, a metric associated with clinical progression,^31^ revealing faster rates of decline in patients with a worse risk profile compared to those with a better risk profile (*p* < 0.0001; Wald test, Table 2). More specifically, with every unit increase in the prognostic risk profile, the rate of decline in the VMI increased by −0.008 per month (95% CI: −0.012 to 0.005). For instance, when comparing two patients with a risk profile score of −6 and −2, the first patient average monthly decline in VMI would be 0.022 units, which would be 0.054 units for the second patient. Results were similar within each sub-cohort (Supplementary Table S2). This relationship is further illustrated in Fig. 2a, showing significant differences between the patient-specific rates of decline in VMI for different prognostic risk groups (*p* = 0.010; one-way ANOVA). The findings of the sex-disaggregated analysis are described in Supplementary Table S3. Of note, comparing VMI scores across days revealed that on Sundays, patients had an average VMI score that was 0.041 (95% CI: 0.067–0.016, *p* = 0.0015; likelihood ratio test) points lower (or a decrease of 2.3%) compared to other days of the week. Thus, there was a small but systematic ‘Sunday effect’.

**Table 2:** Longitudinal trajectory of VMI and its relationship with survival hazard.

Longitudinal submodel	Coefficient	95% CI	*p* value
Time (months)	−0.070	−0.090 to −0.050	<0.0001
Risk profile	0.020	−0.025 to 0.065	0.38
Time (months) × Risk profile	−0.008	−0.012 to 0.005	<0.0001
**Survival submodel**	**Hazard ratio**	**95% CI**	***p* value**
Risk profile	2.24	1.57–3.18	<0.0001
VMI—current value	0.20	0.09–0.44	<0.0001

The results are based on the joint-modelling framework, which combines a linear mixed effects submodel with a survival model. As such, one can estimate the longitudinal trajectory of VMI and its relation with survival hazard while adjusting for (informative) missing data. *p* values based on the Wald test (n = 97).

Abs. VMI, Vertical Movement Index.

### Relationship between rate of decline and survival hazard

**Fig. 3:**
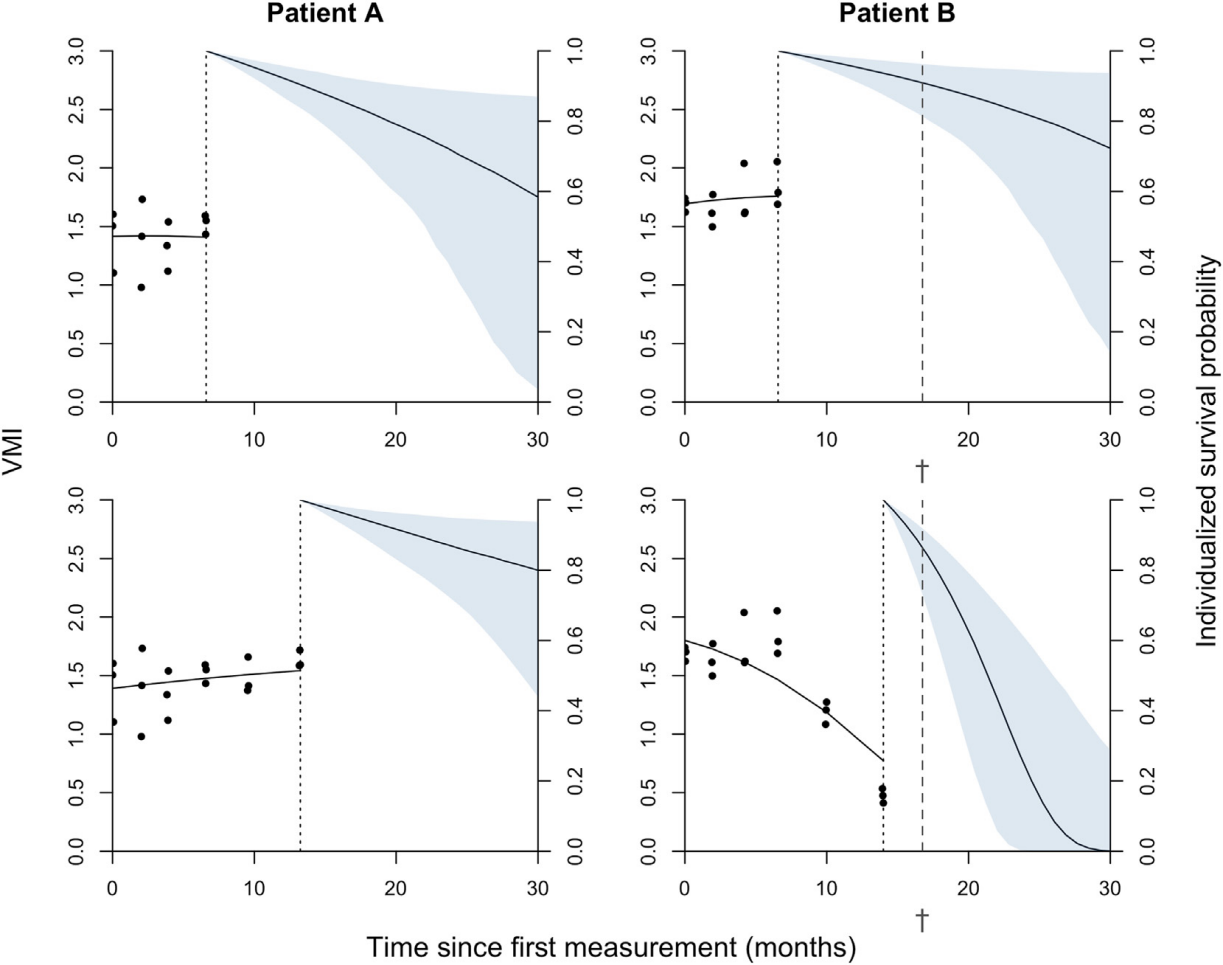
Survival predictions using VMI. Dynamic predictions of the probability of survival for two patients (patient A-B) based on the accumulated data available at that time. The left part of each panel shows the observed longitudinal trajectory of the VMI; the right part shows the estimated survival probability (95% CI). The lower panels illustrate how the probability of survival is dynamically updated as more VMI data become available. Patient B died during follow-up around month 16 (†). Abs. VMI, Vertical Movement Index.

The relationship between baseline VMI and overall survival is illustrated in Fig. 2b, demonstrating that patients who had a lower baseline VMI also experienced a significantly lower probability of survival compared to those who had a higher baseline VMI (HR 2.57, 95% CI: 1.23–5.35, *p* = 0.0092; log-rank test). In a time-varying model – accounting for the change in VMI over time (Table 2)—the current value of VMI was strongly associated with the hazard for death (HR 0.20, 95% CI: 0.09–0.44, *p <* 0.0001; Wald test), increasing the hazard five-fold with every unit decrease. These findings were consistent across cohorts (Supplementary Table S2). The results of the sex-disaggregated analysis are reported in Supplementary Table S3. To illustrate how longitudinal changes in the VMI affect the survival probability of patients, dynamic predictions of the two example patients are presented in Fig. 3. For both patients, two survival predictions are shown using the accumulated VMI data available until month 6. As can be seen, both patients are predicted to have a similar probability of survival for the subsequent months. These survival probabilities change, however, when additional VMI measurements become available, reflecting the dynamic nature of the prediction. In this case, patient B declines rapidly according to the VMI, correctly reflecting the decreased probability of survival during the next few months. The relationship of the ALSFRS-R total and subdomain scores with overall survival are reported in Supplementary Table S4. Current values of both the total score and all subdomain scores were strongly associated with the hazard for death (HR ranging from 0.78 [95% CI: 0.67–0.90, *p* = 0.00093; Wald test] for every unit decrease in the gross subdomain score to 0.92 [95% CI: 0.89–0.95, *p* < 0.0001; Wald test] for every unit decrease in the total score).

### Clinical utility

**Fig. 4:**
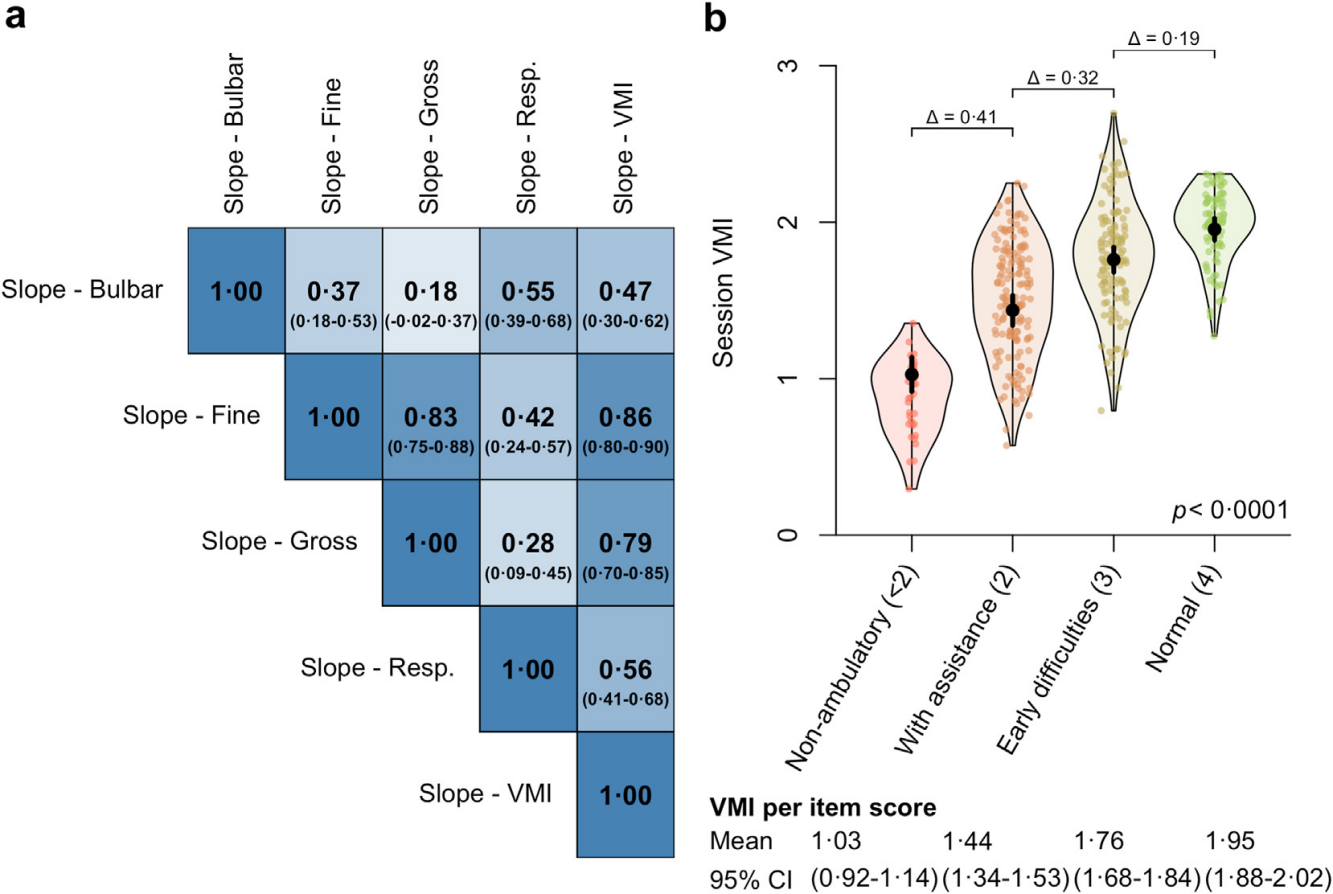
VMI relationship with patient-reported functionality. A correlation matrix of the changes in VMI and the ALSFRS-R subdomain scores to illustrate their relationship; estimates are derived from a multivariate linear mixed effects model (n = 97) and contrasted using Pearson correlation analysis (95% CI) (**a**). In addition, the relationship between VMI and the patient's ambulatory status based on item eight of the ALSFRS-R is presented. *p* value based on the likelihood ratio test (n = 97) (**b**). Abs. ALSFRS-R, ALS Functional Rating Scale, VMI, Vertical Movement Index.

To further explore the clinical utility of VMI, we compared the average VMI to the score of the ALSFRS-R of the same session. The multivariate relationship between changes in VMI and the ALSFRS-R subdomains is presented in Fig. 4a, revealing high correlations of VMI with the fine motor domain (Pearson's r 0.86, 95% CI: 0.80–0.90) and gross motor subdomain (Pearson's r 0.79, 95% CI: 0.70–0.85). Fig. 4b illustrates the relationship between VMI and the patient's ability to walk (item eight ALSFRS-R). As can be seen, the VMI was significantly different (*p* < 0.0001; likelihood ratio test) across ALSFRS-R functional scores. More specifically, when comparing each score with its lower subsequent score, the VMI decreased by 0.19 (95% CI: 0.11–0.28) for “normal” vs. “early difficulties”, 0.32 (95% CI: 0.22–0.43) for “early difficulties” vs. “with assistance”, and 0.41 (95% CI: 0.29–0.52) for “with assistance” vs. “non-ambulatory.”

### Implications for study design

**Fig. 5:**
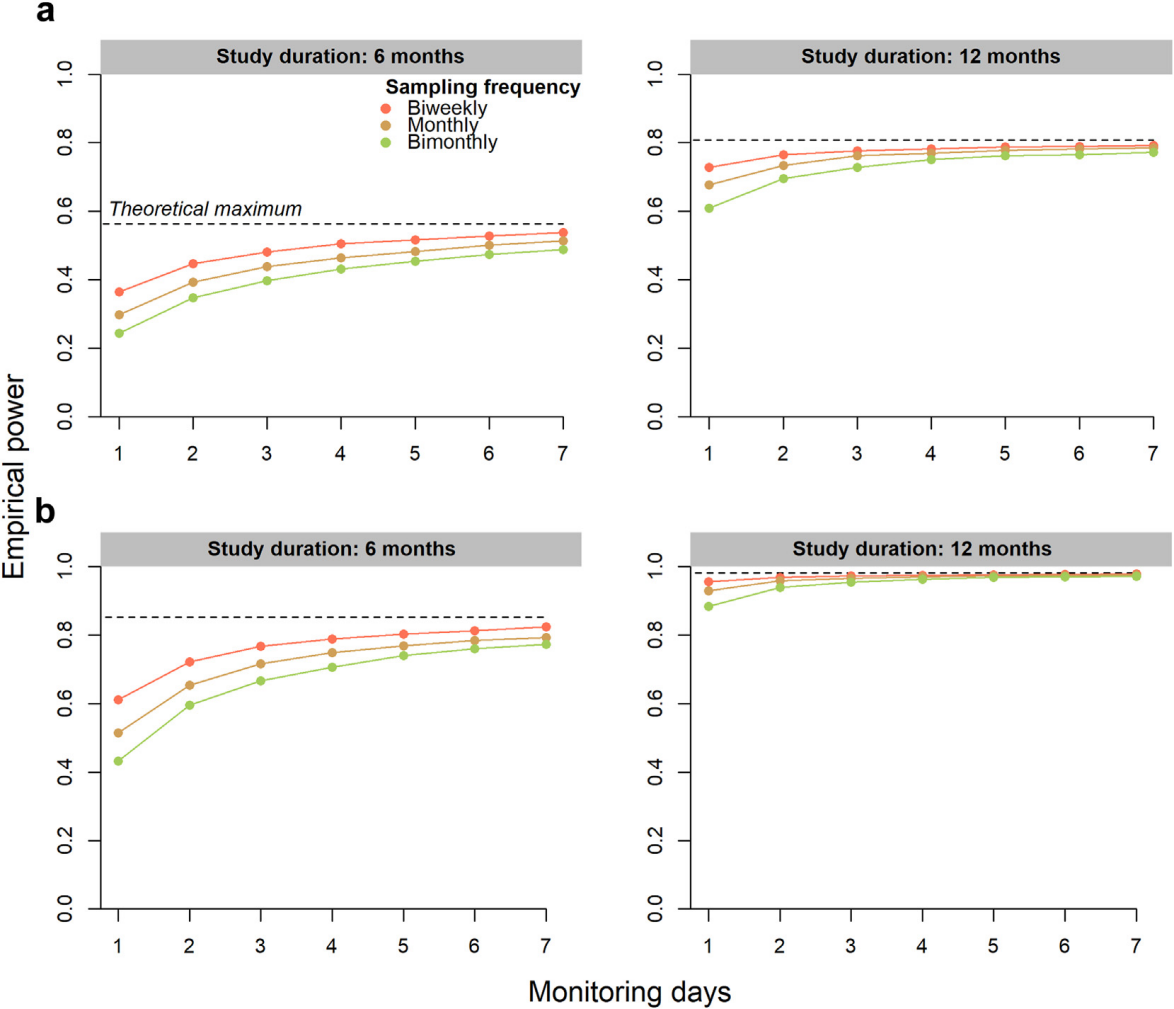
Power estimates for VMI. Based on the parameter estimates derived from a typical clinical trial cohort (risk profile score −6.0 to −2.0; n = 69), we simulated clinical datasets and varied the sampling frequency and monitoring period for different study durations and sample sizes (**a and b**). For each scenario (10,000 simulations), we evaluated whether differential progression in VMI could be detected, based on the time by risk profile interaction term using the likelihood ratio test, and counted the number of simulations that resulted in a significant interaction term (empirical power).

Lastly, to translate findings to future clinical trial design, we evaluated the impact of the monitoring scheme on empirical power. As such, we defined a typical clinical trial cohort based on the risk profile,^40^ thereby excluding 28 patients. Based on the remaining cohort (Supplementary Table S5), we explored the impact of various monitoring schemes on statistical power for different follow-up durations and sample sizes (Fig. 5). As can be seen, more frequent assessments and longer monitoring periods resulted in higher statistical power. This gain in power of more frequent assessments decreased, however, as a function of study duration and sample size. For a study with a 6-month follow-up period, a monthly sampling frequency—each with a 7-day monitoring period—would require 50 patients to detect differential VMI progression rates with 80% power. However, sample size can be reduced by 50% if the follow-up period were to be prolonged to 12 months. A complete list of the simulation parameters is reported in Supplementary Table S6.

## Discussion

In this study, we have shown that an accelerometer-derived outcome, based on passively collected data using wearable sensors in patients with ALS, was highly discriminatory for future disease progression rates and strongly associated with overall survival time. These findings do not only contribute to the growing body of evidence demonstrating the longitudinal association between accelerometer-derived outcomes and conventional ALS disease progression measures, but also support their capability to disentangle and quantify clinical heterogeneity among patients with ALS. These characteristics serve as a motivation to incorporate accelerometery-derived outcomes in clinical trials. Their uptake will be essential for further validation of these outcomes, particularly regarding the relationship with disease-modifying interventions, thereby exploring their potential as candidate surrogate markers, and help to determine meaningful effect sizes.

Advances in digital health technology have enabled the increasing use of digital tools in clinical trials.^41^ Passive monitoring using wearable accelerometers allows for an objective, detailed, and near-continuous collection of data in the home setting, potentially enhancing the detection of domain-specific treatment effects. Furthermore, these technologies provide the opportunity to alleviate the burden of participating in trials by reducing the required number of in-clinic visits, and improve enrolment rates by increasing study accessibility.^42^ Ultimately, these benefits might offer a less burdensome, decentralised, and more patient-focused trial design, holding great promise for drug development.^43^

Although mostly studied as a proof-of-concept in ALS,^14^ previous research has shown the feasibility of accelerometery, demonstrating significant correlations with the ALSFRS-R.^19^, ^20^, ^21^, ^22^, ^23^, ^24^ This study additionally delineated the relationship between the longitudinal trajectory of VMI and overall survival, and demonstrated its ability to differentiate between fast- and slow-progressing patients. This holds significance as it aids in interpreting effect sizes in terms of clinical relevance, thereby contributing to the translation of accelerometer-derived markers to meaningful outcomes.^17^^,^^18^ In addition, as accelerometer-derived measures are not directly relatable to ‘tangible’ daily concepts, additional information is needed to relate them to meaningful patient experience.^44^ By relating the VMI to the symptoms reported by the patient, this study identified potential meaningful differences that could serve as relevant effect sizes in future studies. Nevertheless, to ultimately prove the value for clinical trials, and explore its potential as a surrogate marker, one has to establish whether the clinical effect of a drug (e.g., on ALSFRS-R or survival) is sufficiently reflected in the VMI.^17^^,^^18^^,^^45^ With a view to care, it could be worthwhile to distinguish normal from abnormal VMI values to inform patients about looming clinical milestones, such as the need for assistive devices including walking aids or wheelchair dependency. This would require normative values for the VMI, preferably in a sex, age, and education-matched cohort of non-ALS controls.

The benefit of VMI is that it is conceptually easy to understand, applicable without the need for complex statistical methods, and derivable from any hip-worn device measuring the tri-axial accelerations. A potential disadvantage of this metric, however, is its reliance on hip-worn accelerometery, which has some disadvantages in wear comfort compared to, for example, wrist-worn accelerometery.^46^ On the other hand, wrist-worn accelerometery has its own limitations, including the additional variability it introduces and its potential lower sensitivity for longitudinal changes.^21^ To mitigate this increased variability, one option would be to intensify the monitoring scheme, as shown in Fig. 5, which might be more feasible and less burdensome for wrist-worn accelerometery. Hence, it is of interest to evaluate the trade-off between increased monitoring burden and greater wear comfort in future settings, while acknowledging the additional variability that may arise due to behavioural changes between days and across seasons. In this regard, it might be preferable to implement a monitoring period of at least seven days to average out within-patient variability stemming from differences in daily routines. Of note, previous research showed that wrist-worn accelerometers were better correlated with functional loss in fine motor function, while hip-worn based outcome measures were more related to changes in gross motor function.^21^^,^^23^ Interestingly, this study revealed strong correlations between changes in VMI and functional loss in both fine and gross motor function. Although this finding is potentially an artefact of the domain definition,^9^^,^^10^^,^^47^ it is possibly also attributable to hip-worn sensors not solely capturing lower extremity movement (e.g., turning of the upper chest might result in slight hip movement). An important consideration remains, therefore, to determine whether a uniform metric can be derived that measures the same disease concept when utilizing either hip- or wrist-worn accelerometery.

Our study has several limitations. First, our study utilised the baseline risk profile score to describe ALS heterogeneity. Although this multivariable score comprises seven predictors of disease progression rate,^31^ the inclusion of additional known predictors, such as genetic factors or neurofilament levels,^48^ would likely have enhanced our quantification of ALS heterogeneity. This would decrease the degree of unexplained disease progression, thus leaving less effect to be explained by VMI. As such, the estimation VMI values would probably be lower. Furthermore, variations in cognitive function^49^ or symptoms of anxiety or depression over time may have influenced our estimates of the VMI trajectory and its relationship with overall survival time. In this regard, the association between VMI and survival time was expressed as a HR, which inherently carries the potential of selection bias that may have influenced the results of this study.^50^ Second, we were unable to investigate whether VMI relates to conventional outcome measures that assess domain-specific motor function, such as muscle strength, or to key clinical milestones, such as the use of assistive devices. Although there is a strong association between VMI and the motor domains of the ALSFRS-R,^19^ which was also supported by our findings, the results of additional clinical studies, comparing accelerometery with, for example, (remote) muscle strength measurements,^51^ would further facilitate the interpretation of accelerometer-derived outcomes. Furthermore, the assessment of other domains that may be affected, such as speech and vital capacity, as well as patient-reported outcomes (PROMs), such as global measures of change or quality of life, would enable additional comparisons with accepted measures of ‘ground truth’, which are essential to facilitate clinical interpretation and to determine meaningful changes.^17^^,^^18^^,^^44^ Third, although this study provides important insights into the potential of accelerometer-derived outcomes in clinical trials, the limited sample size prevents the thorough examination of the sensitivity or VMI in non-ambulatory patients or during periods when short-term functional improvements might occur (e.g., initiation of assistive devices). The latter could be valuable in demonstrating the ability of these outcomes to measure positive changes in functionality despite the current lack of disease-modifying treatments. Fourth, our current simulation approach exploring the influence of different monitoring periods on statistical power was primarily based on fixed parameters, which may affect the results if these parameters are considerably different. This approach can be improved by sampling from a probability distribution around each parameter, thus providing more realistic estimates for future settings.^52^ Fifth, this study was performed solely in the Dutch population. We anticipate more challenges regarding the use of accelerometery in regions with limited access to the internet or where the use of telehealth might be less common; also, patients with a lower ‘digital literacy’ level may encounter more problems; this may be an important source of selection for decentralised studies in general.^53^ Sixth, while motor function often exhibits the most rapid progression during a typical trial,^5^^,^^11^ it does not fully encompass the ALS phenotype. To fully capture disease progression, accelerometery measurements should be supplemented with additional outcomes that can be assessed with remote monitoring tools,^51^^,^^54^^,^^55^ evaluating multiple domains that may be affected.

In conclusion, we have shown that an outcome derived from passive hip-worn accelerometery was highly discriminatory in disentangling differential disease progression rates and strongly associated with overall survival time, supporting its use as an outcome measure for ALS clinical trials. Accelerometer-derived outcomes should, however, be obtained in parallel with conventional primary outcomes during clinical trials, in order to further validate these markers as potentially meaningful candidate surrogate endpoints.

## Contributors

J.U., L.B., R.E. contributed to study concept and design. M.M., M.J., contributed to data collection. J.U., R.E. contributed to data analysis. J.U., R.E. contributed to drafting the manuscript and figures. All authors contributed to reviewing and editing the manuscript. All authors read and approved the final version of the manuscript. J.U. and R.E. have directly accessed and verified the underlying data reported in the manuscript.

## Data sharing statement

De-identified individual participant data are available on DataverseNL repository (https://doi.org/10.34894/QPFHWV).

## Declaration of interests

Nothing to report.
