# Supplementary material for: Remote monitoring of amyotrophic lateral sclerosis using wearable sensors detects differences in disease progression and survival: a prospective cohort study
Source: eBioMedicine. 2024 Apr 6;103:105104. doi: 10.1016/j.ebiom.2024.105104 (PMC11004066; doi:10.1016/j.ebiom.2024.105104)
Supplement: Supplementary Tables S1–S6 [file mmc1.docx]

**SUPPLEMENTARY**

| **Table of content** |  |
| --- | --- |
| **Content** | **Page** |
| Table 1. Study population and design settings of each cohort | 2 |
| Table 2. Longitudinal trajectory of VMI and its relationship with survival hazard for each cohort study | 3 |
| Table 3. Longitudinal trajectory of VMI and its relationship with survival hazard for each sex | 4 |
| Table 4. Relationship between ALSFRS-R and survival hazard | 5 |
| Table 5. Longitudinal trajectory of VMI and its relationship with survival hazard for the clinical trial cohort | 6 |
| Table 6. Parameter estimates used to simulate a clinical dataset | 7 |

| **Table 1. Study population and design settings of each cohort** | | |
| --- | --- | --- |
|  | **Cohort 1** | **Cohort 2** |
| **Study population** |  |  |
| Inclusion criteria | - Diagnosis of ALS according to the El Escorial criteria for definite, probable (laboratory supported) or possible ALS, or the diagnosis of progressive muscular atrophy (PMA) or primary lateral sclerosis (PLS) | - 18 years or older - Diagnosis of ALS according to the El Escorial criteria for definite, probable (laboratory supported) or possible ALS - Absence of any neurological symptom associated with ALS |
| Exclusion criteria |  | - Inability to lie in supine position for one hour - Having a tracheostomy or other assisted ventilation - Presence of intellectual disability or mental illness - Asymptomatic carriers of ALS-related genes with human immunodeficiency virus, vitamin B5 toxicity, or peripheral mononeuropathy |
| **Design settings** |  |  |
| Maximum follow-up duration (months) | 18 | 24 |
| Measurement frequency (months) | 2 - 3 | 3 |
| Accelerometer device | ActiGraph GT9X Link | ActiGraph GT9X Link |
| Accelerometer wear location | Right hip during waking hours | Right hip during waking hours |
| Accelerometer wear period (days) | 3 - 7 | 7 |
| Accelerometer sampling frequency (Hz) | 30 | 30 |
| Accelerometer collection method | Part of the main study | Optional, as part of a larger study |
| ALSFRS-R collection method | Remote | In-clinic |
| Survival status collection method | Online nationwide population registry | Online nationwide population registry |
| Abs. ALSFRS-R = ALS Functional Rating Scale - Revised | | |

| **Table 2. Longitudinal trajectory of VMI and its relationship with survival hazard for each cohort study** | | | |
| --- | --- | --- | --- |
|  | **Cohort 1 (n = 42)** | | |
| **Longitudinal submodel** | **Coefficient** | **95% CI** | ***p* value** |
| Time (months) | -0·067 | -0·095 to -0·040 | < 0·0001 |
| Risk profile | 0·028 | -0·034 to 0·090 | 0·38 |
| Time (months) × Risk profile | -0·007 | -0·012 to -0·002 | 0·0033 |
| **Survival submodel** | **Hazard ratio** | **95% CI** | ***p* value** |
| Risk profile | 2·60 | 1·37 to 4·95 | 0·0035 |
| VMI – current value | 0·21 | 0·07 to 0·62 | 0·0047 |
|  | **Cohort 2 (n = 55)** | | |
| **Longitudinal submodel** | **Coefficient** | **95% CI** | ***p* value** |
| Time (months) | -0·067 | -0·091 to -0·043 | < 0·0001 |
| Risk profile | 0·010 | -0·050 to 0·071 | 0·74 |
| Time (months) × Risk profile | -0·009 | -0·014 to -0·005 | < 0·0001 |
| **Survival submodel** | **Hazard ratio** | **95% CI** | ***p* value** |
| Risk profile | 2·06 | 1·32 to 3·21 | 0·0015 |
| VMI – current value | 0·22 | 0·07 to 0·67 | 0·0077 |
| *p* values based on the Wald test  Abs. VMI = Vertical Movement Index | | | |

| **Table 3. Longitudinal trajectory of VMI and its relationship with survival hazard for each sex** | | | |
| --- | --- | --- | --- |
|  | **Male (n = 68)** | | |
| **Longitudinal submodel** | **Coefficient** | **95% CI** | ***p* value** |
| Time (months) | -0·073 | -0·098 to -0·048 | < 0·0001 |
| Risk profile | 0·045 | -0·004 to 0·095 | 0·073 |
| Time (months) × Risk profile | -0·009 | -0·014 to -0·005 | < 0·0001 |
| **Survival submodel** | **Hazard ratio** | **95% CI** | ***p* value** |
| Risk profile | 2·63 | 1·61 to 4·30 | 0·00012 |
| VMI – current value | 0·12 | 0·04 to 0·38 | 0·00033 |
|  | **Female (n = 29)** | | |
| **Longitudinal submodel** | **Coefficient** | **95% CI** | ***p* value** |
| Time (months) | -0·058 | -0·090 to -0·026 | 0·00033 |
| Risk profile | -0·035 | -0·130 to 0·061 | 0·48 |
| Time (months) × Risk profile | -0·006 | -0·011 to -0·001 | 0·016 |
| **Survival submodel** | **Hazard ratio** | **95% CI** | ***p* value** |
| Risk profile | 2·18 | 1·28 to 3·70 | 0·0039 |
| VMI – current value | 0·45 | 0·14 to 1·43 | 0·18 |
| *p* values based on the Wald test  Abs. VMI = Vertical Movement Index | | | |

| **Table 4. Relationship between ALSFRS-R and survival hazard** | | | |
| --- | --- | --- | --- |
| **ALSFRS-R**^a^ | **Hazard ratio** | **95% CI** | ***p* value** |
| Total score | 0·92 | 0·89 - 0·95 | <0·0001 |
| Bulbar subdomain | 0·86 | 0·78 - 0·94 | 0·00079 |
| Fine subdomain | 0·86 | 0·78 - 0·96 | 0·0082 |
| Gross subdomain | 0·78 | 0·67 - 0·90 | 0·00093 |
| Respiratory subdomain | 0·85 | 0·76 - 0·95 | 0·0038 |
| *p* values based on the Wald test  Abs. ALSFRS-R = ALS Functional Rating Scale - Revised  ^a^ Each outcome was fitted separately using the current value as the association structure | | | |

| **Table 5. Longitudinal trajectory of VMI and its relationship with survival hazard for the clinical trial cohort^a^** | | | |
| --- | --- | --- | --- |
| **Longitudinal submodel** | **Coefficient** | **95% CI** | ***p* value** |
| Time (months) | -0·095 | -0·139 to -0·051 | < 0·0001 |
| Risk profile | 0·024 | -0·082 to 0·130 | 0·66 |
| Time (months) × Risk profile | -0·014 | -0·024 to -0·005 | 0·0035 |
| **Survival submodel** | **Hazard ratio** | **95% CI** | ***p* value** |
| Risk profile | 2·76 | 1·70 to 4·48 | < 0·0001 |
| VMI – current value | 0·22 | 0·10 to 0·49 | 0·00022 |
| *p* values based on the Wald test  Abs. VMI = Vertical Movement Index  ^a^ Clinical trial cohort defined as having a risk profile score -6·0 to -2·0 (both inclusive) | | | |

| **Table 6. Parameter estimates used to simulate a clinical dataset** | | |
| --- | --- | --- |
|  | **Clinical trial cohort^a^** | ***p* value** |
| **Data description** |  |  |
| Number of patients | 69 | - |
| Number of observations | 1,258 | - |
| Number of events | 28 | - |
| **Longitudinal process** |  |  |
| Random effects |  |  |
| Variance intercept (σ^2^_1_) | 0·384 | - |
| Variance slope (σ^2^_2_) | 0·047 | - |
| Variance slope^2^ (σ^2^_3_) | 0·003 | - |
| Covariance intercept – slope (σ^2^_1,2_) | 0·042 | - |
| Covariance intercept – slope^2^ (σ^2^_1,3_) | -0·055 | - |
| Covariance slope – slope^2^ (σ^2^_2,3_) | -0·865 | - |
| Residual variance (σ^2^_e_) | 0·199 | - |
| Fixed effects |  |  |
| Intercept (β0) | 1·807 | <0·0001 |
| Slope (β1) | -0·095 | <0·0001 |
| Risk profile (β2) | 0·024 | 0·66 |
| Slope x risk profile | -0·014 | 0·0035 |
| **Time-to-event process** |  |  |
| Weibull intercept (γ^0^) | 0·795 | 0·55 |
| Risk profile score (γ^1^) | 1·014 | <0·0001 |
| Association parameter (ρ) | -1·499 | 0·00022 |
| Weibull shape parameter (α) | 0·376 | 0·058 |
| *p* values based on the Wald test  ^a^ Clinical trial cohort defined as having a risk profile score -6·0 to -2·0 (both inclusive) | | |
